# Sanfu herbal patch applied at acupoints in patients with bronchial asthma: statistical analysis plan for a randomised controlled trial

**DOI:** 10.1186/s13063-022-06990-7

**Published:** 2022-12-20

**Authors:** Danghan Xu, Jialing Li, Anqi Di, Peidan Yang, Xiaoyan Xie, Yiming Xu, Jun He

**Affiliations:** 1 The First Affiliated Hospital of Guangzhou University of Chinese Medicine, Guangzhou, 510405 China; 2 The Second Clinical College, Guangzhou University of Chinese Medicine, Guangzhou, 510006 China; 3 Guangdong Provincial Hospital of Chinese Medicine, 510120 Guangzhou, China; 4 School of Basic Medicine, Guangzhou Medical University, Guangzhou, 511436 China

**Keywords:** Bronchial asthma, Sanfu herbal patch, Statistical analysis plan

## Abstract

**Background:**

Sanfu herbal patch (SHP) is widely used in the prevention and treatment of bronchial asthma in China, but its efficacy and mechanism of action are not completely clear. This trial aims to determine the efficacy of SHP and the underlying mechanism.

**Methods/design:**

We will conduct a multi-centre parallel randomised controlled trial consisting of 72 participants with bronchial asthma recruited and randomly allocated at a ratio of 1:1 into two groups. The patients in one group will receive three courses of SHP treatment, and the patients in the other group will receive placebo treatment, with 24 weeks of follow-up evaluation for both groups. The primary outcome, i.e. forced expiratory volume in the first second (FEV1), which refers to the change in FEV1 from the beginning of the baseline to the end of 3 treatment sessions (TSs), will be assessed and compared via Student’s *t* test or the Mann–Whitney *U* test. Other outcomes will include questionnaire surveys and laboratory indicators. Detailed and complete statistical analyses in a double-blinded fashion will be provided for evaluating this trial.

**Discussion:**

The data we obtain will be examined based on the above statistical analysis, which will help to reduce the risk of external reporting bias and data-driven results.

**Trial registration:**

Chinese Clinical Trial Registry (http://www.chictr.org.cn), ChiCTR1900024616. Registered on 19 July 2019.

**Supplementary Information:**

The online version contains supplementary material available at 10.1186/s13063-022-06990-7.

## Background

Bronchial asthma (BA), one of the most common chronic non-communicable diseases, is characterized by airway inflammation, airway stenosis, and airway hyper-responsiveness [1]. The main clinical manifestations of asthma are recurrent chest tightness, wheezing, shortness of breath, and cough. Persistence of these symptoms without any relief can cause irreversible airway narrowing and remodelling and even death [2]. At present, approximately 4.3% of people worldwide are affected by asthma [3]. Moreover, the global prevalence of asthma in adults continues to increase [4, 5], and it is predicted that approximately 5.7% of the global population will be affected in the future. There are approximately 30 million asthma patients in China [6], and with the acceleration of urbanisation, the incidence of asthma in China is gradually increasing [7].

At present, asthma treatment consists of anti-inflammatory and anti-spasmodic strategies, with the aim of controlling symptoms, reducing recurrence, and improving daily quality of life. Drug treatment includes glucocorticoids and β2 receptor agonists, but there are many adverse reactions, and repeated use will lead to drug resistance [8]. Therefore, safe and effective treatments that can relieve the symptoms of asthma are urgently needed.

Sanfu herbal patch (SHP) is one of the most important external therapies for the treatment of respiratory diseases in traditional Chinese medicine [9, 10]. Indeed, SHP is the most commonly used traditional Chinese therapy for asthma in China [9] and can effectively reduce the frequency of asthma and improve patient’s quality of life [11, 12]; it has advantages of being painless, non-invasive, safe, effective and economical. SHP reduces the number of helper T lymphocyte 2 (Th2) in patients with asthma and can increase the helper T lymphocyte 1 (Th1)/helper T lymphocyte 2 (Th2) ratio, which reduces the inflammatory response in patients with allergic asthma [13]. However, there is currently a lack of large-sample multi-centre clinical studies to prove the clinical efficacy of SHP, and the underlying mechanism has not yet been fully elucidated. We designed this trial to determine the clinical treatment effect in patients with BA after three courses of SHP treatment and 24 weeks of follow-up evaluation. The trial will also provide data that will enable us to determine whether SHP decreases airway inflammation and reverses bronchoconstriction.

The trial protocol was previously published [14], with additional detailed information on the trial principles, qualification criteria and intervention measures. The purpose of this statistical analysis plan is to detail and prespecify how the data collected in this trial will be used and analysed to minimise reporting deviation and make the test as transparent as possible. The statistical analysis protocol completed under double-blind conditions was approved (version 1.0) by the First Affiliated Hospital of Guangzhou University of Traditional Chinese Medicine on November 1, 2020.

## Methods/design

### Study design

**Fig. 1:**
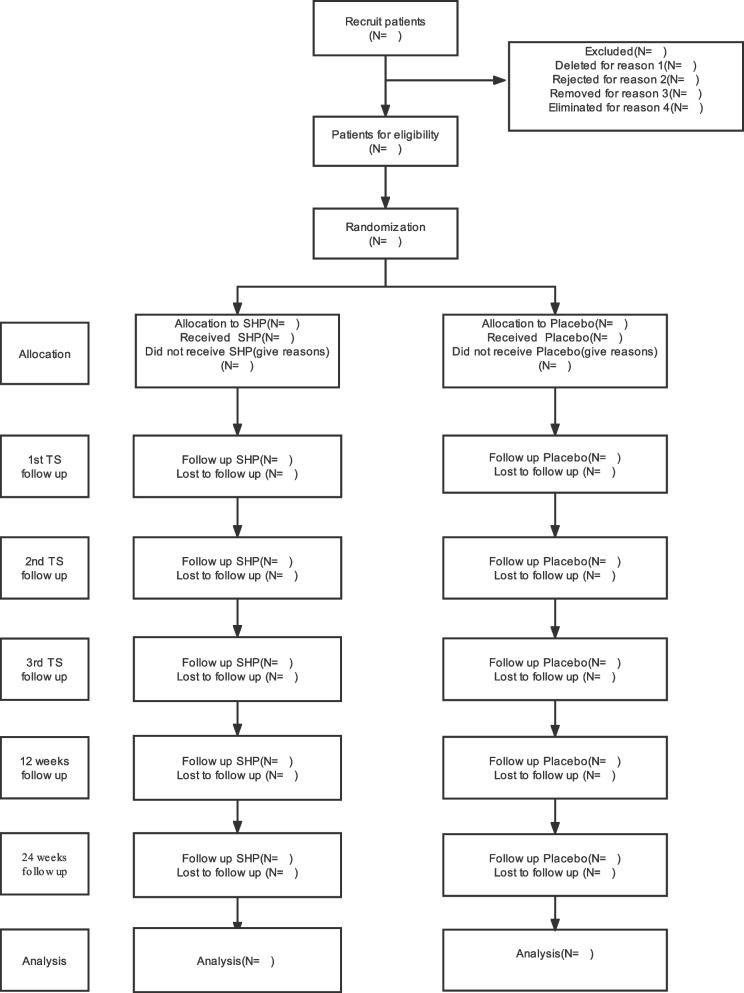
Flow diagram. SHP, Sanfu herbal patch; TS, treatment session; 1st TS: the 1st treatment during the first 10 days. 2nd TS: the 2nd treatment during the second 10 days. 3rd TS: the 3rd treatment during the third 10 days. 12 weeks: the first follow-up period from baseline. 24 weeks: the second follow-up period from baseline

**Fig. 2:**
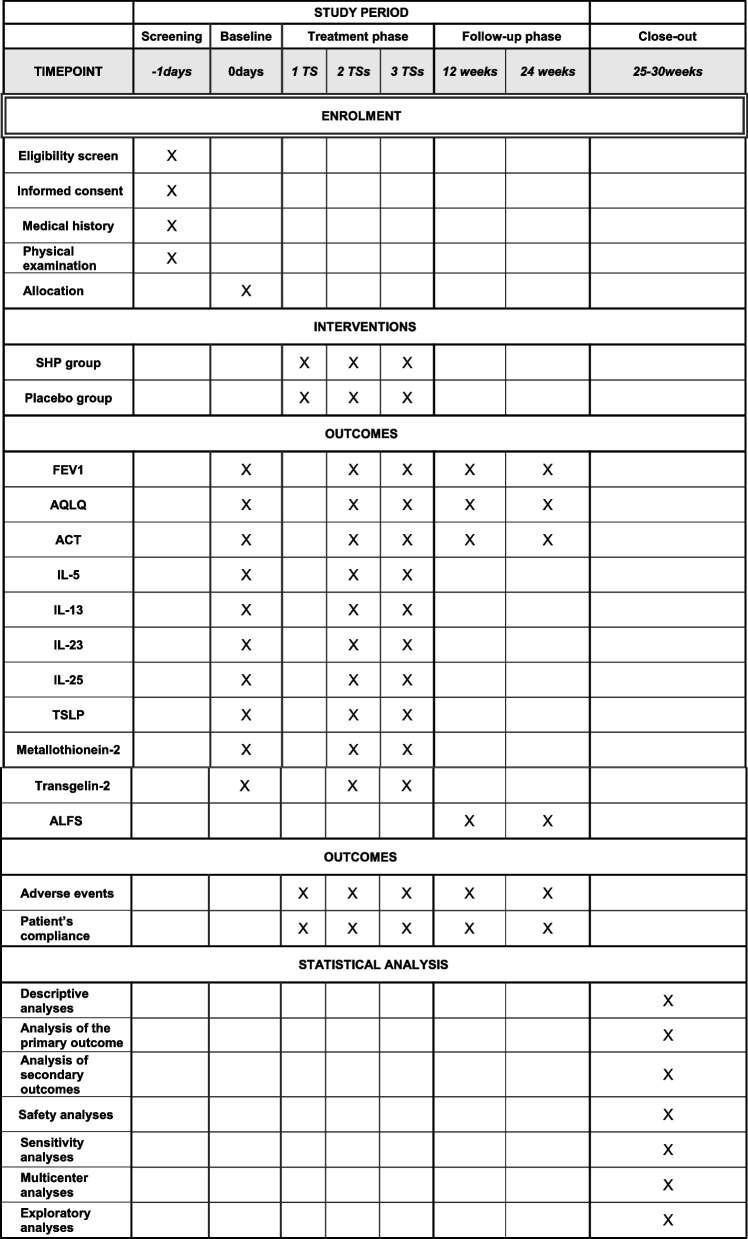
Study schedule. 1 TS: the 1st treatment during the first 10 days. 2 TS: the 2nd treatment during the second 10 days. 3TS: the 3rd treatment during the third 10 days. 12 weeks: the first follow-up period from baseline. 24 weeks: the second follow-up period from baseline. 25–30 weeks: the period of statistical analysis from baseline. All outcomes will be assessed at baseline, the end of 2 TSs, and the end of 3 TSs during treatment

This study is a multi-centre parallel randomised controlled trial. We will screen BA participants according to rigid inclusion and exclusion standards. The specific standards have been described in detail in the published protocol [14]. Randomisation will be performed by Strategy Applications (SAS, version 9.2, SAS Institute, Inc., Cary, USA) by generating random permuted blocks. The randomisation process will be implemented by the Key Unit of Methodology in Clinical Research in Guangdong Provincial Hospital of Chinese Medicine. An independent researcher will prepare a treatment card with a serial number and one of two group numbers. Groups 1 and 2 will represent the SHP and placebo groups, respectively. The researcher will label the tubes “group 1” or “group 2.” Participants will receive a treatment card from the independent researcher to ensure blindness to the treatment assignment results [14]. After obtaining the consent of the participants, we will randomly assign 72 eligible subjects (1:1) to the SHP group or the placebo group, and they will receive three courses of SHP treatment or placebo treatment, respectively, and then 24 weeks of follow-up evaluation (Figs. 1 and 2).

Throughout the trial, only independent researchers will be aware of the treatment assignments, whereas the participants, acupuncturists, nurses, data managers and statisticians will be blinded to the treatment assignments. All staff will be unblinded at the end of the study. The operating assistants will prepare identical-looking SHP patches and placebo patches using tubes marked with the same names on the treatment cards. In addition, the acupuncturists, operating assistants and nurses will be asked not to communicate their possible assignments with the participants, and the participants will have to wait in the room for 120 minutes and remove the patches while facing away from the nurses [14].

The trial has been previously registered in the Chinese Clinical Trial Registry (ChiCTR1900024616, registered on July 19, 2019).

### Objectives

To determine the clinical efficacy of SHP in the treatment of BA and the underlying mechanism.

## Outcomes

The outcomes of this trial include the change in forced expiratory volume in the first second (FEV1) from the beginning of baseline to the end of 3 treatment sessions (TSs) as the primary outcome; the questionnaire survey and laboratory indicators will be secondary outcomes. Although the specific details have been explained in the published protocol [14], we briefly discuss them here.

### Primary outcome

The primary outcome will be the change in FEV1 from the beginning of baseline to the end of 3 TSs, which will be included in the analysis. The FEV1 test will be conducted in the pulmonary function examination room of the First Affiliated Hospital through the pulmonary function test system (Masterscreen, Carefusion Germany 234 GmbH) at the time of baseline measurement and at the end of 3 treatment courses. It should be noted that none of the participants will use bronchodilators during the measurement. We generally consider that pulmonary insufficiency occurs when the FEV1 value is less than 80% of the expected value [15].

### Secondary outcome

#### Questionnaire survey

As secondary outcomes, we will ask participants to complete the Asthma Quality of Life Questionnaire (AQLQ) [16] and the Asthma Control Test (ACT) [17] at the second and third TSs at the 12th and 24th weeks compared with baseline. The raw outcomes will be used in the statistical analysis.

Asthma Quality of Life Questionnaire (AQLQ): There are 35 items, and the score ranges from 1 point (completely restricted) to 7 points (unrestricted), reflecting the quality of life of adult asthma patients. The lower the score is, the more severe the restrictions on the patient’s quality of life is.

Asthma Control Test (ACT): There are 5 items of Patient-Reported Outcome Measurement, with a total score of 5 to 25; it is used to assess asthma control in asthma patients [18]. The lower the score is, the greater the deficiency in asthma control is.

In addition, we will ask the participants to complete the Asthma Long-term Follow-up Scale (ALFS) [19] in the 12th and 24th weeks of follow-up to evaluate treatment efficacy in each group. ALFS includes four aspects, frequency and severity of symptoms, time of onset, FEV1 and drug use (name, dose, and date); its use will help us to judge the effect of SHP treatment.

#### Laboratory indicators

We will assess levels of airway inflammatory factors (IL-5, IL-13, IL-23, IL-25 and TSLP) and tracheal smooth muscle cell regulatory proteins (metallothionein-2 and transgelin-2) during the baseline examination and at the end of the second and third TSs.

### Safety outcome

We will record adverse events throughout the trial and classify them as regards their relationship to SHP treatment.

### Sample size

The sample size was calculated based on the primary outcome measure by PASS 11.0 (Power Analysis and Sample Size, NCSS, LLC., Kaysville, UT, USA). The calculated power was set at 90%, and the two-sided confidence interval was set at 95%. The target difference in this study is improvement by 15%. Previous studies have shown an FEV1 score for the SHP group of 79.41 ± 13.87 and for the placebo group of 59.61 ± 11.52 [20]. Considering that *α* = 0.05, *β* = 0.1, and an attrition rate of 15%, 36 cases per group were calculated based on two-sided 95% confidence intervals.

### Statistical analysis

#### Principles of statistical analysis

In this study, *p* < 0.05 will be considered statistically significant. The process involved in this study will follow the CONSORT reporting guidance (http://www.consort-statement.org/) to produce a CONSORT flow diagram (Fig. 1). Two independent staff members will complete the data entry, and a third party will complete the inspection and storage. All statistical analyses will be performed by two statisticians using the PASW Statistic 24.0 software (IBM SPSS, Inc., Armonk, NY, USA). The statistical results will be independently submitted to the primary investigator by the two statisticians, who will not be allowed to communicate with each other. We will use this method to maintain consistency with blinding and will do our best to reduce bias related to human factors.

In this analysis, the results of the primary outcome will determine the conclusion of the study. The analysis of secondary outcomes and the exploratory analysis are intended to supplement the primary outcome. Intention-to-treat will be used unless specified otherwise.

#### Descriptive analyses

In this study, we will collect baseline patient sociodemographic characteristics including age, gender, ethnicity, body mass index (BMI), education level, employment status, marital status, economic level, smoking history, and alcohol consumption history. When the above is missing, data imputations will not be conducted. All data will be described by different methods according to their type and distribution status. For continuous variables, we will use the mean ± standard deviation to describe data in a normal distribution; the median and interquartile range (IQR) are more applicable when the data show a skewed distribution. Percentages will be used to present data for categorical variables. The data presentation is shown in Table 1.

**Table 1:** Baseline characteristics

Characteristics	Sanfu herbal patch (***n*** = )	Placebo (***n*** = )
Age, years		
Gender		
Male		
Female		
Race (%)		
Han		
Minorities		
Educational level (%)		
Primary education or less		
Secondary education		
Tertiary education		
Marital status		
Married		
Unmarried		
BMI, kg/m^2^		
Employment status		
On duty		
Laid off		
Retired		
Smoking		
Yes		
No		
Drinking		
Yes		
No		
FEV1(L)		
AQLQ		
ACT		
IL-5 (pg/ml)		
IL-13 (pg/ml)		
IL-23 (pg/ml)		
IL-25 (pg/ml)		
TSLP (pg/ml)		
Metallothionein-2 (ng/ml)		
Transgelin-2 (ng/ml)		
ALFS		

*FEV1* forced expiratory volume in the first second, *AQLQ* Asthma Quality of Life Questionnaire, *ACT* Asthma Control Test, *IL-5* interleukin 5, *IL-13* interleukin 13, *IL-23* interleukin 23, *IL-25* interleukin 25, *TSLP* thymic stromal lymphopoietin, *ALFS* Asthma Long-term Follow-up Scale

For continuous variables, we will use the mean ± standard deviation to describe data in a normal distribution; the median and interquartile range (IQR) are more applicable when the data show a skewed distribution. Percentages will be used to present data for categorical variables

#### Analysis of the primary outcome

For the primary outcome, we will all report means, differences in means, confidence intervals and *p*-values. The primary outcome will be assessed and compared via Student’s *t* test or the Mann–Whitney *U* test. First, we will use a histogram to test whether the data distribution satisfies a normal distribution. An *F*-test will also be used to determine whether the data are homogeneous. Based on the results of the test, we will use the *t* test if the data are normally distributed and show homogeneity of variance; if they are not, we will use the Mann–Whitney *U* test. Missing data should be imputed. In addition, we will perform linear regression analysis for the change in FEV1 to improve power and precision, which will use intervention as independent variables. The data presentation is indicated in Table 2.

**Table 2:** Primary and secondary outcomes

Outcomes	Sanfu herbal patch (***n*** = )	Placebo (***n*** = )	SHP vs placebo Difference (95% CI)	***p***-value
FEV1, L				
1stTS				
2ndTS				
3rdTS				
12 weeks follow-up				
24 weeks follow-up				
AQLQ				
2ndTS				
3rdTS				
12 weeks follow-up				
24 weeks follow-up				
ACT				
2ndTS				
3rdTS				
12 weeks follow-up				
24 weeks follow-up				
IL-5, pg/ml				
2ndTS				
3rdTS				
IL-13, pg/ml				
2ndTS				
3rdTS				
IL-23, pg/ml				
2ndTS				
3rdTS				
IL-25, pg/ml				
2ndTS				
3rdTS				
Metallothionein-2, ng/ml				
2ndTS				
3rdTS				
Transgelin-2, ng/ml				
2ndTS				
3rdTS				
ALFS				
12 weeks follow-up				
24 weeks follow-up				

*SHP* Sanfu herbal patch, *FEV1* forced expiratory volume in the first second, *AQLQ* Asthma Quality of Life Questionnaire, *ACT* Asthma Control Test, *IL-5* interleukin 5, *IL-13* interleukin 13, *IL-23* interleukin 23, *IL-25* interleukin 25, *ALFS* Asthma Long-term Follow-up Scale, *TS* treatment session

Because FEV1 will be measured at baseline, 2nd TS, 3rd TS, 12th week, and 24th week, the mean data at each time point will be plotted as a line graph. As a line chart is used to present the change trend, a hypothetical test will not be performed. If the graph displays a non-linear relationship, we will use curve fitting to explore whether a threshold saturation effect is present.

#### Analysis of secondary outcomes

We will report the AQLQ and ACT total and item scores separately at the second and third TSs and at the 12th and 24th weeks and the ALFS total and item scores separately at the 12th and 24th weeks of follow-up. First, we will use a histogram to test whether the data distribution satisfies the normal distribution. To assess homogeneity of variance, we will examine residuals by producing diagnostic plots of residuals against fitted values. If the assumption of normality and constant variance are not met, we will consider using data transformations.

Some laboratory indicators will also be used, including IL-5, IL-13, TSLP, IL-23, IL-25, TSLP, metallothionein-2, and transgelin-2. These experimental metrics will be reported at the end of the second and third TSs. The above data are continuous variables, and the statistical analysis method will be the same as described above. For secondary outcomes, we will report means, differences in means, confidence intervals, and *p*-values using raw data.

#### Analysis of missing data

Due to the application of intention-to-treat, we will impute missing data to ensure all participants are included in the primary analysis in the group to which they were randomised. For missing data in the primary analysis, multiple imputation will be applicable in this study. Although multiple imputation has incomparable advantages over simple imputation, there are also some disadvantages, such as the inability to distinguish whether observed data are missing in a random fashion [21]. Therefore, the practical process of various interpolations needs to be carefully designed. This imputation will be used in the primary analysis. The observation data will be calculated by age, gender, drinking, smoking and body mass index.

Based on the suggestions of Rubin [22], we will create five copies of the data set. The Monte Carlo Markov chain method will be applied in multiple imputation. In these five copies, missing values will be replaced by estimated values, which we will use for the statistical analysis. In the second step, we will use a multiple regression model to combine the results of the analysis in the first step with the standard error calculated using Rubin’s rules to obtain the effect size we need. However, the above method is only applicable in the case of a normal distribution. When faced with non-normally distributed data, we will require statisticians using transformations to an approximate normal before estimation and then using transformations to an original scale after completing the estimation.

All imputations will be based on missing data as missing completely at random or missing at random. Therefore, if the statistician believes the data are not randomly missing, the result should be noted in the statistical report. When reporting results, statisticians should follow the reporting principle of epidemiological multiple imputation [23].

#### Safety analyses

This study will evaluate the safety of SHP through the incidence of adverse events. First, we will report the number of adverse events and calculate the incidence of adverse events in the SHP and placebo groups. Second, we will use Fisher’s test to compare the proportion of participants reporting at least one adverse event. Finally, we will analyse factors related to the occurrence of adverse events to guide our next work.

#### Sensitivity analyses

Sensitivity analysis is relevant to the primary outcome. This statistical analysis will consist of two parts. First, we will use the principle of intention-to-treat for analysis of outcomes to reduce likelihood of false positives. Second, we will use per-protocol to perform a second analysis of the data of the primary outcomes and use this as sensitivity analysis. Because sensitivity analysis will be employed to test the robustness of the primary analysis in the presence of noncompliance.

Those who meet the following criteria will be excluded from the per-protocol analysis: (1) receiving other complementary treatments, such as moxibustion, acupuncture and massage; (2) using a drug not planned for the study, such as herbal medicine and dipropionate; (3) no available outcome measurement records.

If the results of the sensitivity analysis differ from the results of the intention-to-treat analysis, the researcher will repeatedly discuss this matter with the statistician to cautiously and accurately interpret the clinical significance of the data.

When reporting the test results, we will report both at the same time to avoid publication deviation. The use of multiple interpolation may cause some unexpected errors [24–26]. We will use complete case analysis as sensitivity analyses for data imputations.

#### Multi-centre analyses

Although we have adopted a variety of methods to ensure the consistency of the trial at different centres, centre effects may still occur. Therefore, it is necessary to consider the centre effect in the statistical analysis. We will test the primary outcome for an interaction with the centre, and we will perform regression models to test the interaction. The model fit with and without interaction will be compared, and the *p*-value of the interaction term will be reported. If there is an interaction, relevant reasons will be determined to explain it. If there are no interactions, we will further evaluate whether there is a central effect. The central effect will be deducted if there is a central effect. In general, problems can be solved by constructing a mixed-effects model and using centre as a random factor.

#### Exploratory analyses

We will consider using some statistical methods for exploratory research to guide our next research design. We will carry out a series of subgroup analyses to identify dominant SHP groups. The pathogenesis of bronchial asthma is very complicated, and we will collect many indicators in our study that are highly specific to its pathogenesis. Therefore, we will carry out subgroup analysis to determine which population may be the advantage population.

According to our clinical experience, we believe that higher BMI (≥ 30), female gender and older age (≥ 52 years) may be the advantage population for SHP for bronchial asthma. Then, we will perform subgroup analysis for age (18 ≤ age < 52, ≥ 52 years), gender (male or female) and BMI (< 30 or ≥ 30).

It should be noted that this type of analysis is data-driven and is very prone to false positives. Therefore, researchers should be cautious when interpreting the trial’s clinical significance. The results of this subgroup analysis may not be presented in the public manuscript but used only by the research team as a reference direction for designing the next project.

## Discussion and trial status

The results of this trial will determine whether there is evidence that SHP can reduce the clinical symptoms of BA patients and improve their quality of life. In addition, we will assess tracheal smooth muscle cell regulatory proteins (metallothionein-2 and transgelin-2) and airway inflammatory factors (IL-5, IL-13, IL-23, IL-25, and TSLP) to elucidate whether the treatment mechanism occurs through reducing airway inflammatory factors and reversing bronchoconstriction.

Here, we provide a detailed and complete statistical analysis plan to evaluate the current trial, which will assist in reducing the risk of external reporting bias and data-driven results. This article was compiled according to “Guide to the Content of Statistical Analysis Programs.” As of December 2019, 72 patients from 4 centres were randomly assigned; follow-up actions will be completed by December 2023. The analysis plan was prepared before the completion of the experimental data collection phase.

## Trial status

The participants are beginning to be recruited for this study (protocol version 2.0, 19 July 2019). The study will be conducted from May 2020 to December 2023.

## Supplementary Information

**Additional file 1.**

## Data Availability

The data from this randomised controlled study are unavailable at the time of publication. Individual participant data are available upon request.

## Abbreviations

BA: Bronchial asthma
SHP: Sanfu herbal patch
TS: Treatment session
FEV1: Forced expiratory volume in the first second
AQLQ: Asthma Quality of Life Questionnaire
ACT: Asthma Control Test
ALFS: Asthma Long-term Follow-up Scale
TSLP: Thymic stromal lymphopoietin
BMI: Body mass index
IL-5: Interleukin 5
IL-13: Interleukin 13
IL-23: Interleukin 23
IL-25: Interleukin 25

## References

[1] Yang L, Guo YS, Jiang JQ, Guo XJ, Xu YP, Tian Y, Xiong Y, Han L. The effect of stimuli on basophil-mediated atopic responses during asthmatic lying-in women and in newborns. Hybridoma (Larchmt). 2012;31(4):255–61. 10.1089/hyb.2012.0024. 10.1089/hyb.2012.0024 PMC3420553 22894778

[2] Alvarez GG, Schulzer M, Jung D, Fitzgerald JM (2005). A systematic review of risk factors associated with near-fatal and fatal asthma. Can Respir J.

[3] Papi A, Brightling C, Pedersen SE, Reddel HK (2018). Asthma. Lancet..

[4] Vos T, Flaxman AD, Naghavi M (2012). Years lived with disability (YLDs) for 1160 sequelae of 289 diseases and injuries 1990–2010: a systematic analysis for the Global Burden of Disease Study 2010. Lancet.

[5] Anandan C, Nurmatov U, van Schayck OC, Sheikh A. Is the prevalence of asthma declining? Systematic review of epidemiological studies. Allergy. 2010;65(2):152-67. 10.1111/j.1398-9995.2009.02244.x. 10.1111/j.1398-9995.2009.02244.x 19912154

[6] Bateman ED, Hurd SS, Barnes PJ, Bousquet J, Drazen JM, FitzGerald JM, Gibson P, Ohta K, O'Byrne P, Pedersen SE, Pizzichini E, Sullivan SD, Wenzel SE, Zar HJ. Global strategy for asthma management and prevention: GINA executive summary. Eur Respir J. 2008;31(1):143-78. 10.1183/09031936.00138707. Erratum in: Eur Respir J. 2018;51(2): PMID: 18166595. 10.1183/09031936.00138707 18166595

[7] Chen Y, Wong GW, Li J. Environmental Exposure and Genetic Predisposition as Risk Factors for Asthma in China. Allergy Asthma Immunol Res. 2016;8(2):92-100. 10.4168/aair.2016.8.2.92. 10.4168/aair.2016.8.2.92 PMC4713885 26739401

[8] Kozian A, Schilling T, Hachenberg T. Asthma bronchiale - Anästhesiologisches Management [Anesthetic management in bronchial asthma]. Anasthesiol Intensivmed Notfallmed Schmerzther. 2016;51(6):402-9. 10.1055/s-0041-106371. 10.1055/s-0041-106371 27359239

[9] Zhou F, Yang D, Lu JY, Li YF, Gao KY, Zhou YJ (2015). Characteristics of clinical studies of summer acupoint herbal patching: a bibliometric analysis. BMC Complement Altern Med.

[10] Yang XC, Yin T, Gao Q, Kong LJ. The immunomodulatory effect of acupoint application for childhood asthma: a systematic review and meta-analysis. Evid Based Complement Alternat Med. 2015;2015:896247. 10.1155/2015/896247. 10.1155/2015/896247 PMC4426892 26000027

[11] Xiaqiu W, Jin P, Guoqin L, Wei Z, Guangxia L, Baoyan L (2015). Efficacy evaluation of summer acupoint application treatment on asthma patients: a two-year follow-up clinical study. J Tradit Chin Med.

[12] RanBang M, HyunKim J, YeonMin S (2015). Clinical effectiveness of the Sanfu herbal patch at acupoints for respiratory diseases including otitis media in children: a pilot before-and-after study. Eur J Integr Med.

[13] Zhao SM, Wang HS, Zhang C, Hu J, Zhuang LL, Wang X, et al. Repeated herbal acupoint sticking relieved the recurrence of allergic asthma by regulating the Th1/Th2 cell balance in the peripheral blood. Biomed Res Int. 2020:1879640. 10.1155/2020/1879640 PMID: 32509851; PMCID: PMC7251437. 10.1155/2020/1879640 PMC7251437 32509851

[14] Xie X, Xu D, Zhuang L, Liu H, Tan S, Lu Y (2020). Sanfu herbal patch applied at acupoints in patients with bronchial asthma: study protocol for a randomized controlled trial. Trials..

[15] Zheng JP, Gao Y (2010). Practical guide to pulmonary function testing.

[16] Everhart RS, Smyth JM, Santuzzi AM, Fiese BH (2010). Validation of the Asthma Quality of Life Questionnaire with momentary assessments of symptoms and functional limitations in patient daily life. Respir Care.

[17] Hasegawa T, Koya T, Sakagami T, Kagamu H, Arakawa M, Gejyo F (2013). The Asthma Control Test, Japanese Version (ACT-J) as a predictor of Global Initiative for Asthma (GINA) guideline-defined asthma control: analysis of a questionnaire-based survey. Allergol Int.

[18] Ramakrishnan K, Lee LK, Safioti G (2019). Asthma Control Test (ACT) scores correlate with health-related quality of life (HRQoL) in patients with asthma. J Allergy Clin Immunol.

[19] Zarqa A, Glattre DC, Suppli UC (2013). Long-term mortality among adults with asthma. Chest..

[20] Lu YM. Summary of 35 cases of relief period of bronchial asthma treated with Sanfu acupoint patch. Hunan. J Tradit Chin Med. 2011. 10.16808/j.cnki.issn1003-7705.2011.03.009 In Chinese.

[21] Sterne JA, White IR, Carlin JB (2009). Multiple imputation for missing data in epidemiological and clinical research: potential and pitfalls. BMJ..

[22] Rubin DB (1987). Multiple imputation for nonresponse in surveys.

[23] Klebanoff MA, Cole SR (2008). Use of multiple imputation in the epidemiologic literature. Am J Epidemiol.

[24] Hippisley-Cox J, Coupland C, Vinogradova Y, Robson J, May M, Brindle P (2007). Derivation and validation of QRISK, a new cardiovascular disease risk score for the United Kingdom: prospective open cohort study. BMJ.

[25] Peto R. Doubts about QRISK score: total/HDL cholesterol should be important [electronic response to Hippisley-Cox J, et al]. BMJ. 2007; www.bmj.com/cgi/eletters/335/7611/136#172067.

[26] Hippisley-Cox J, Coupland C, Vinogradova Y, Robson J, May M, Brindle P. QRISK— authors’ response [electronicresponse]. BMJ. 2007; www.bmj.com/cgi/eletters/335/7611/136#174181.

