# Supplementary material for: Sanfu herbal patch applied at acupoints in patients with bronchial asthma: statistical analysis plan for a randomised controlled trial
Source: Trials. 2022 Dec 20;23:1025. doi: 10.1186/s13063-022-06990-7 (PMC9764530; doi:10.1186/s13063-022-06990-7)
Supplement: Supplementary file 1 — Additional file 1. [file 13063_2022_6990_MOESM1_ESM.pdf]

广州中医药大学第一附属医院
The First Affiliated Hospital of Guangzhou University of Chinese Medicine

Ethical Approval Letter

NO. ZYYECK【2019】049

<table>
<tr><td>Protocol Title</td><td colspan="3">Cinical standardized research and mechanism research on the prevention and treatment of bronchial asthma based on the theory of treating winter disease in summer</td></tr>
<tr><td>Objective</td><td colspan="3">□Phase I clinical trial;□Phase II clinical trial; □Phase III clinical trial;□Phase IV clinical trial;□Bioequivalence test;□Clinical trial of import drug registration;
□Reevaluation of drugs on the market;□Experiment on protective varieties of traditional Chinese Medicine; ■scientific research;□Clinical trial of medical instruments; □Clinical verification of medical devices; □postgraduate education;
□ Other</td></tr>
<tr><td>Department /Specialties</td><td>Acupucture</td><td>Principal Investigator</td><td>Jun He</td></tr>
<tr><td>Approved Documents</td><td>1.Application for Ethical Review;
2.Study Protocol(Ver2.0);
3.Informed Consent Form(Ver2.0);
4.Resume of Principle Investigator and GCP Cetification;
5.Case Report Form(V5.27, 20190527);
6.Declaration of Conflict of Interest.</td><td>Sponsor</td><td>The First Affiliated Hospital of Guangzhou University of TCM</td></tr>
<tr><td>Review modes</td><td>Mode of the initial review:
□Expedited review
■Full Board Review(Date:20-06-2019)</td><td>Decision</td><td>Final decision: Approve</td></tr>
<tr><td>Annual Follow-Up Review Frequency</td><td>12 months (please submit annual / periodic follow-up review documents 1 month before 1July 2020)</td><td>Expiry of Approval Letter</td><td>1 year（If the research project
has not been launched within 1 year, please resubmit the initial ethical review application）</td></tr>
<tr><td>Attentions</td><td colspan="3">According to the Approaches to Ethical Review of Biomedical Research Involving Human Beings(2016,China), the SFDA Good Clinical Practice for Medical Devices(2016, China), theGuiding Principles for Ethical Review of Drug Clinical Trials (2010, China), WMA Declaration of Helsinki, and International Ethical Guidelines for Biomedical Research Involving Human Subjects (CIOMS), It is agreed to carry out this clinical research after ethicalreview.
After the research completed, please submit the conclusive report. Serious adverse events, protocol violation, suspension/termination of the research and unexpected events affecting the risk-to-benefit ratio of research shall be reported to the committee in a</td></tr>
</table>

	timely manner.<br>If the study protocol and informed consent form have been modified or the main researcher changed, the ethics committee shall be notified in time. The research shall be reviewed again and execute after approved by the committee.
**Contact/ EC Member& Secretary**	Xinying Li<br>Tel:020-36588667 or 020-36591965<br>Fax:020-36591346<br>Email:gztcmlunli@163.com
**Signature of the Chair/ Authorized Person**	Date:01-07-2019

Statement: The Ethics Committee organizes and works in strict accordance with Chinese GCP and related laws and regulations.

广州中医药大学第一附属医院伦理委员会
